# Synovialosarcome de la glande parotide: à propos d’un cas

**DOI:** 10.11604/pamj.2026.53.110.47187

**Published:** 2026-03-03

**Authors:** Zakaria El Hafi, Abderrazak El Bouraqadi, Razika Bencheikh, Mohamed Anass Benbouzid, Inssaf El Moumni, Amine Lachgar, Leila Essakalli

**Affiliations:** 1 ENT-Head and Neck Surgery Department, Hospital of Specialities, CHU Ibn Sina, Rabat, Morocco,; 2 Faculty of Medicine and Pharmacy, Mohamed V University, Rabat, Morocco,; 3 Radiotherapy Department, Hospital of Oncology, CHU Ibn Sina, Rabat, Morocco

**Keywords:** Synovialosarcome, glande parotide, radiothérapie, cas clinique, Synovial sarcoma, parotid gland, radiotherapy, case report

## Abstract

Le synovialosarcome de la glande parotide constitue une entité pathologique extrêmement rare, se présentant comme une forme particulière de sarcome synovial de la région cervico-faciale. Cette localisation atypique, liée à l'absence de tissu synovial dans cette région, complique fréquemment le diagnostic différentiel, notamment avec des néoplasmes mésenchymateux primaires ou secondaires, ainsi que des myoépithéliomes. Nous rapportons le cas d'un patient âgé de 27 ans présentant une masse résiduelle de la glande parotide gauche apparue 6 ans après une parotidectomie exofaciale pour un synovialosarcome confirmé. Ce travail explore les implications cliniques, pathologiques et thérapeutiques, tout en synthétisant les données récentes de la littérature.

## Abstract (en)

Synovial sarcoma of the parotid gland is an extremely rare pathological entity, presenting as a particular form of synovial sarcoma of the cervicofacial region. This atypical localisation, linked to the absence of synovial tissue in this region, frequently complicates the differential diagnosis, notably with primary or secondary mesenchymal neoplasms and myoepitheliomas. We report the case of a 27-year-old patient presenting with a residual mass of the left parotid gland that appeared 6 years after exofacial parotidectomy for a confirmed synovial sarcoma. This work explores the clinical, pathological and therapeutic implications, while synthesising recent data from the literature.

## Introduction

Le synovialosarcome (SS) est une tumeur mésenchymateuse agressive, représentant 2,5 à 3,5% des sarcomes localisés dans la tête et le cou, et seulement 0,1% des cancers affectant cette région [1,2]. Localisée majoritairement dans les extrémités, cette tumeur apparaît rarement dans la région parotidienne, où elle représente un défi diagnostique en raison de son caractère mimétique de lésions bénignes ou d'autres tumeurs malignes salivaires. Contrairement à ce que suggère son nom, la forme cervico-céphalique ne dérive pas de cellules synoviales matures, mais plutôt de cellules mésenchymateuses pluripotentes. La prise en charge optimale repose sur une approche multidisciplinaire intégrant une chirurgie large et, lorsque nécessaire, des traitements adjuvants. En raison de leur rareté dans la région de la tête et du cou, les cas cervico-céphaliques sont généralement rapportés sous forme de cas cliniques isolés ou de séries de cas restreintes.

## Patient et observation

**Information relative au patient:** il s'agit d'un patient âgé de 27 ans, opéré il y a 7 ans pour une tumeur parotidienne gauche, ayant bénéficié d'une parotidectomie exofaciale. Le résultat anatomopathologique était en faveur d'une prolifération tumorale indifférenciée de cellules fusiformes disposées en nodules de dimension variable séparés par une épaisse bande hyaline paucicellulaire, avec une activité mitotique modérée, sans nécrose mais avec des marges tumorales mal limitées. Ce dernier a été affiné par une étude immunohistochimique qui a confirmé le diagnostic du synovialosarcome de grade II (anticorps anti-EMA de positivité focale). Le patient n'a pas reçu de radio ni de chimiothérapie adjuvante (perdu de vue).

**Résultats cliniques:** actuellement le patient présente une réapparition de la masse parotidienne gauche qui augmente progressivement de taille sans paralysie faciale ni limitation de l'ouverture buccale ni autres signes oto-rhino-laryngologiques.

**Figure 1:**
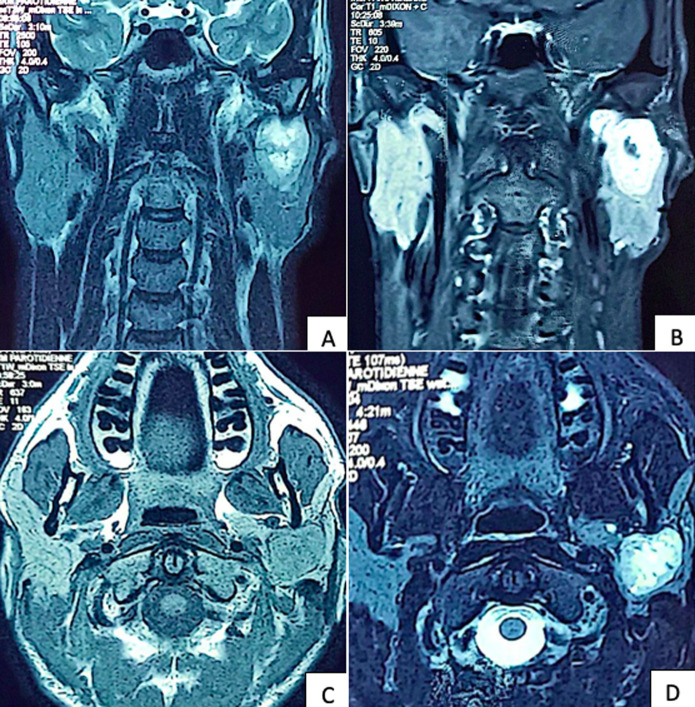
IRM parotidienne: A) coupe coronale séquence T2, B) coupe coronale séquence T1 injectée, C) coupe axiale séquence T1, D) coupe axiale séquence T2 fat saturation, montrant une glande parotide gauche de taille normale et aux contours réguliers

**Démarche diagnostique:** une imagerie par résonance magnétique (IRM), cervico-parotidienne (Figure 1), objectivant une lésion nodulaire située à la jonction des portions superficielles et profondes de la glande parotide. Cette lésion, de forme ovoïde et bien limitée, apparaît en hyposignal T1, en hypersignal T2, avec une restriction de diffusion associée à une diminution du signal sur la carte du coefficient apparent de diffusion. Ses dimensions sont estimées à 22 × 23 x 34 mm, sans atteinte ganglionnaire cervicale.

**Intervention thérapeutique:** le patient a bénéficié d'une exérèse totale du reste de la parotide nodulaire englobant le nerf facial qui était disséqué et préservé, et l'étude anatomopathologique donne le même diagnostic que l'ancienne intervention qui est un synovialosarcome de grade II. Cette fois, le patient n'est pas perdu de vue, il a eu un complément par radiothérapie en suivant un protocole à visée curative type *volumetric modulated arc therapy* dont le volume cible: volume clinique microscopique contenant la loge parotidienne et le lit tumoral (CTV T) est de 66 Gy en 33 fractions, 2 Gy/fraction, CTV N (aires prophylactiques cervicales) II et III 54 Gy en 33 fractions, 1,6 Gy/fr.

**Suivi et résultats:** le patient est suivi à ce jour, 5 mois après la chirurgie, et 2 mois après la fin des séances de radiothérapie, sans découverte de récidive cliniquement ni d’apparition de complication de radiothérapie ou de paralysie faciale.

**Perspective du patient:** le patient est globalement satisfait de la prise en charge chirurgicale et radiothérapique dont il a bénéficié. Il rapporte avoir bien vécu cette seconde intervention, se disant rassuré par la préservation de sa fonction faciale et par l'absence, à ce jour, de séquelle ou de complication. Conscient d'avoir été perdu de vue après sa première chirurgie, il insiste sur l'importance qu'il accorde désormais à un suivi médical régulier et exprime sa confiance envers l'équipe multidisciplinaire qui l'a pris en charge tout au long de son parcours de soins.

**Consentement éclairé:** un consentement éclairé écrit, daté et signé a été obtenu du patient.

## Discussion

Le SS est une tumeur maligne de haut grade représentant 2,5 à 3,5% des sarcomes localisés dans la région cervico-céphalique et seulement 0,1% des cancers affectant cette région anatomique [2,3]. Comparativement aux synovialosarcomes d'autres localisations, les formes touchant la tête et le cou sont considérées comme présentant un potentiel accru de métastases locorégionales et systémiques, le mode de dissémination dominant étant hématogène [2,4]. Il se classe comme la quatrième forme la plus fréquente après l'histiocytome fibreux malin, le liposarcome et le rhabdomyosarcome. Cette tumeur survient principalement chez les jeunes adultes, avec une légère prédominance masculine. Bien que les données sur le pronostic des SS cervico-céphaliques soient limitées, il est souvent présumé par les cliniciens qu'il est plus sombre que celui des SS primaires sur d'autres sites anatomiques [5].

Son diagnostic repose sur une approche multidisciplinaire alliant imagerie, histopathologie, immunohistochimie et analyse moléculaire. L'IRM, examen de choix, met en évidence un signal typiquement hétérogène en T1 et hyperintense en T2 avec rehaussement post-gadolinium, permettant d'évaluer la taille et l'extension de la tumeur [6]. La cytoponction échoguidée fournit une première évaluation, mais peut être source de confusion avec des tumeurs bénignes. L'examen histopathologique des biopsies révèle des variantes monophasiques (cellules fusiformes), biphasiques (éléments fusiformes et glandulaires) ou peu différenciées, ces dernières étant souvent associées à une agressivité accrue [6,7]. L'immunohistochimie joue un rôle clé, les marqueurs fréquemment exprimés étant la cytokératine, l'antigène de membrane épithéliale (EMA) et la vimentine, tandis que CD34 et S-100 sont négatifs, facilitant la distinction avec d'autres sarcomes [6,8]. La détection spécifique de la translocation t(X;18)(p11;q11), responsable de la fusion des gènes SS18-SSX, par PCR ou FISH, constitue un critère diagnostique majeur pour confirmer le diagnostic [8].

Le traitement repose sur une approche multimodale, avec une priorité donnée à l'exérèse chirurgicale complète, visant à obtenir des marges saines, ce qui constitue le pilier du traitement curatif. Une parotidectomie totale ou radicale est souvent nécessaire, en fonction de l'extension tumorale et de l'implication des structures adjacentes. L'épargne ou le sacrifice du nerf facial dépend de la localisation tumorale et de son infiltration [7,9]. La radiothérapie adjuvante est couramment employée dans les cas présentant des facteurs de mauvais pronostic, notamment des marges chirurgicales positives, un grade histologique élevé, ou une taille tumorale supérieure à 5 cm. Cette stratégie a démontré son efficacité dans le contrôle local de la maladie et l'amélioration des taux de survie globale à 5 ans, atteignant jusqu'à 80% dans certaines séries [1,9,10]. La chimiothérapie, bien que controversée, est réservée aux formes avancées, métastatiques ou récidivantes. Le suivi postopératoire implique une surveillance rigoureuse par imagerie, étant donné le risque élevé de récidive locale ou de métastases à distance, principalement pulmonaires [7]. Dans notre cas, on est à seulement 2 mois après la fin de la radiothérapie, ce qui ne permet pas de juger notre efficacité thérapeutique et de détecter les paramètres à améliorer dans notre attitude de suivi.

Le pronostic des patients atteints de SS de la glande parotide est influencé par plusieurs facteurs, notamment la taille tumorale, les marges chirurgicales, le grade histologique et la présence de métastases. Les tumeurs de petite taille (<5 cm) avec des marges d'exérèse négatives sont associées à un meilleur pronostic, avec une survie globale à 5 ans atteignant 80% dans certaines études [7,9]. En revanche, les tumeurs volumineuses, les marges positives ou les formes monophasées mal différenciées sont corrélées à une augmentation significative des récidives locales et des métastases, réduisant les taux de survie à long terme [1,10].

Les techniques de médecine moléculaire, telles que l'identification des translocations t(X;18), pourraient également aider à surveiller la progression ou la récidive tumorale [9,10]. Malgré les progrès thérapeutiques, le pronostic à long terme reste réservé, avec une survie globale à 10 ans de 34% pour les tumeurs avancées. Les stratégies futures, incluant l'utilisation de thérapies ciblées et d'immunothérapies, offrent des perspectives prometteuses pour améliorer les résultats chez ces patients [1,9].

## Conclusion

Le synovialosarcome de la glande parotide, bien que rare, constitue un défi diagnostique et thérapeutique en raison de son agressivité et de sa présentation atypique. La prise en charge repose sur une chirurgie complète avec des marges saines, associée à des traitements adjuvants tels que la radiothérapie. Le pronostic, influencé par la taille tumorale et les marges, reste réservé, avec un risque élevé de récidives et de métastases. Ce cas souligne l'importance d'une prise en charge multidisciplinaire et d'un suivi rigoureux pour optimiser les résultats cliniques.

## References

[1] Stanbouly D, Litman E, Lee KC, Philipone E (2021). Synovial sarcoma of the head & neck: A review of reported cases in the literature. J Stomatol Oral Maxillofac Surg.

[2] Harb WJ, Luna MA, Patel SR, Ballo MT, Roberts DB, Sturgis EM (2007). Survival in patients with synovial sarcoma of the head and neck: association with tumor location, size, and extension. Head Neck.

[3] Al-Daraji W, Lasota J, Foss R, Miettinen M (2009). Synovial sarcoma involving the head: analysis of 36 cases with predilection to the parotid and temporal regions. Am J Surg Pathol.

[4] Moore DM, Berke GS (1987). Synovial sarcoma of the head and neck. Arch Otolaryngol Head Neck Surg.

[5] Gopalakrishnan V, Amini B, Wagner MJ, Nowell EN, Lazar AJ, Lin PP (2017). Synovial Sarcoma of the Head and Neck: A Single Institution Review. Sarcoma.

[6] Ziani Y, Khalfi L, Choumi F, Boudhas A, Al Bouzidi A, Abouchadi A (2014). Synovialosarcome primitif de la glande parotide [Primary synovial sarcoma of the parotid gland]. Rev Stomatol Chir Maxillofac Chir Orale.

[7] Wushou A, Miao XC (2015). Tumor size predicts prognosis of head and neck synovial cell sarcoma. Oncol Lett.

[8] Rigante M, Visocchi M, Petrone G, Mule A, Bussu F (2011). Synovial sarcoma of the parotid gland: a case report and review of the literature. Acta Otorhinolaryngol Ital.

[9] Colizza A, Di Stadio A, Ralli M, De Luca P, Cavaliere C, Gilardi A (2022). Systematic Review of Parotid Gland Sarcomas: Multi-Variate Analysis of Clinicopathologic Findings, Therapeutic Approaches and Oncological Outcomes That Affect Survival Rate. Cancers (Basel).

[10] Quan H, Sreekissoon S, Wang Y (2023). Synovial sarcoma of the head and neck: A review of reported cases on the clinical characteristics and treatment methods. Front Cell Dev Biol.

